# A Praiseworthy Effort: A Reference Library for Dentists

**Published:** 1894-06

**Authors:** 


A PRAISEWORTHY EFFORT: A REFERENCF LIBRARY FOR DENTISTS.—The need of a reference library has been felt by the dentists of Baltimore, in the preparation of papers for dental society meetings. Several years since a resolution was adopted by the Maryland State Dental Association urging the members to donate dental books or pamphlets to the Peabody Library or the Enoch Pratt Library, so they might be at the command of all the members of the profession. The resolution received no attention until recently, when the newly elected president, Dr. Charles C. Harris, issued a written request for the members to contribute dental works and back numbers of dental journals. The librarian of the Enoch Pratt Library, Dr. Bernard C. Steiner, has not only consented to have the dental monthlies, which may be donated, bound; but in the preparation of a new catalogue, finding that the subject of dentistry is not well represented on the shelves of the library, has also provided ten or fifteen of the standard works on dentistry not usually found in the private library of the practitioner. There is no telling how helpful this effort to aid the practicing dentist and the students of the Baltimore College of Dental Surgery and the University of Maryland Dental Department will be, and it is hoped that a generous response has been made to Dr. Harris's request.

